# Measurement of acute postoperative pain intensity in orthopedic trials: a qualitative concept elicitation study

**DOI:** 10.2340/17453674.2024.42182

**Published:** 2024-11-07

**Authors:** Karen T BJØRNHOLDT, Carina W G ANDERSEN

**Affiliations:** 1 Department of Orthopedic Surgery, Horsens Regional Hospital; 2 Department of Orthopedic Surgery, Svendborg Hospital, Denmark

## Abstract

**Background and purpose:**

Pain intensity is an important outcome in clinical trials of surgery because pain relief is important to patients. Currently, recommended scales are the numeric rating scale 0–10 and visual analogue scale. However, these scales allow for considerable influence of individual imagination, previous experience, and coping skills, limiting proficiency in comparative clinical trials. We aimed to explore postoperative expressions of “how much it hurts”—the first step to improve pain intensity measurement.

**Methods:**

This was a qualitative study using inductive content analysis: words and visual cues describing pain intensity were collected from (i) existing pain intensity measures by search of COSMIN, PubMed, and Google, (ii) patient interviews recorded and transcribed word-for-word, (iii) clinician interviews transcribed likewise, and (iv) 100 patient telephone interviews with notes taken. After familiarization, the collected expressions were labelled inductively in categories and assembled in tables (case and theme-based matrices).

**Results:**

Descriptors fell into 12 categories: intensity (slight/strong), evaluative (negligible/unbearable), cognitive impact (distracting/can be ignored), activity impact (limits some/all activity), sleep impact (can/cannot sleep), examples (like stubbing a toe), physical signs (crying/writhing), associated symptoms (nauseating/tiring), treatment (ice helps/need morphine), affective (annoying/dreadful), discriminative (aching/piercing), and general recovery (hindering recovery/functional interference). Many visual cues were also identified. Literature and recorded interviews gave rise to the categories, and telephone interviews found saturation, providing no further categories.

**Conclusion:**

Pain intensity is expressed by terms that fall into 12 categories and by a variety of graphic elements. This advances development of a patient-reported outcome measure of pain intensity for orthopedic trials.

Pain is an important outcome in trials, as established in many core outcome sets [1]. Pain as a construct consists of multiple domains: the sensory domain (intensity, variation, duration, localization, characteristics such as throbbing), the reactive domain (affective/evaluative components) and impact (physical, cognitive, emotional, social) [2,3]. Surgical interventions specifically target the sensory domain. Generally, we should assess targeted outcomes directly.

Pain is a personal experience [4], and the gold standard of measurement is by self-report when possible [5]. Objective measure of pain being impossible, the quality of pain comparisons in trials depends on the quality of pain communication (measurement). Quality assessment of available measurement tools guided by COSMIN standards [6-8] was recently published for adult postoperative pain, questioning the validity [9]. The most common tools are the numeric rating scale (NRS) and visual analogue scale (VAS) [10]. In these, the anchor of “worst imaginable/possible pain” relies on patients’ imagination and experiences [11]. This impairs comparisons between patients as well as associations of preoperative, acute postoperative, and chronic pain. What is needed is standardized self-report, requiring a scale interpreted accurately by all patients [12], i.e., a reliable understanding of “what is meant by 5.”

Going back to the drawing board, the following specifications are made. The basic premise is that pain intensity can be communicated and compared between patients. The target population is orthopedic patients, able to self-report, read, and understand greater-than/less-than rather than dichotomous thinking, excluding children < 12 years [13] and cognitively disabled. The context of use is self-administered web-based in hospital and after discharge, for evaluating postoperative pain intensity for up to 3 months as an outcome. The primary intention is for randomized clinical trials with repeating measurements.

This papers aims to present the concept elicitation by establishing a library (item bank) of descriptors, a categorized collection of expressions of pain intensity in this population. This is the first step to advance the measurement of acute postoperative pain intensity in orthopedic trials [6,14].

## Methods

The study is based on and is reported according to the COSMIN checklist [6], FDA guidance [15], and review articles of PROM development [16,17].

To develop the semantics of a pain scale, identification of possibly relevant wording was obtained from literature, patient interviews, and clinician interviews. Qualitative methodological assistance was provided by an experienced researcher trained in qualitative research and patient communication.

### Literature

Searches for pain intensity measures were made in the COSMIN database, Google, and PubMed. In the Cosmin database (https://database.cosmin.nl/), the search was made using “Pain” (All fields) and Filters: Adult, patient reported outcome, physical symptom state, questionnaires/interviews/diaries/clinical rating scales, NRS/NRS-11/NRS-21/NRS-Child/PI-NRS (pain intensity NRS). Google.com was searched for images of “pain measure” and “smerteskala” (Danish, meaning pain scale). The PubMed.gov search strategy is given in Table 1. The articles, their references, and images were included if they contained pain intensity described in words, phrases, or visual cues such as color [18] and graphic presentation. Other ways of expressing pain intensity such as sound [19] or finger pressure [20] were excluded for lack of use in a web-based scale. Words describing pain intensity were explored further in English online dictionaries and thesauri to aid understanding. The resulting collection of words and phrases were qualitatively assessed by familiarization and inductively categorized.

**Table 1:** PubMed search strategy for pain intensity measures

Search #	Query
1	“pain measurement/methods”[MeSH Major Topic] OR “checklist”[MeSH Major Topic] OR “Patient Outcome Assessment”[MeSH Major Topic] OR “Self Report”[MeSH Major Topic] OR “Patient Reported Outcome Measures”[MeSH Major Topic]
2	“Pain”[MeSH Major Topic]
3	#1 AND #2
4	#3 NOT animal [Filter]
5	#4 NOT “Mental Disorders”[MeSH Terms]
6	#5 AND (“infant”[MeSH Terms] OR “child”[MeSH Terms]) NOT (“adolescent”[MeSH Terms] OR “adult”[MeSH Terms])
7	#5 NOT #6
8	#7 AND “systematic review”[Filter]
Search for things not (yet) indexed in Medline:	
1	“measure*”[Text Word] OR “self report*”[Text Word] OR “assessment”[Text Word]
2	“pain”[Text Word]
3	((“publisher”[Filter] NOT “pubstatusnihms”[All Fields]) NOT “pubstatuspmcsd”[All Fields]) NOT “pmcbook”[All Fields]
4	#1 AND #2 AND #3
5	#4 NOT “child*”[All Fields]
6	#5 AND (systematicreview[Filter])

### Patient interviews

10 patients were recruited face-to-face in the Day Surgery Centre, Horsens Regional Hospital and interviewed after their surgery. Sample size was 10 based on expected saturation and more than 7 interviews being considered very good in the COSMIN checklist for content validity [6]. Patients were chosen based on availability and provided consent on the day of surgery. For a broader representation, maximum 2 patients for each NRS score were chosen. Interviews were conducted in Danish in the postoperative resting area. Interviews were semi-structured, based on an interview guide (translation in Supplementary data). Semi-structured interviews were chosen to allow for spontaneous use of pain descriptors without specific prompts from the interviewer. Patients were asked to rate their pain on the 0–10 pain scale and then prompted open-endedly to elaborate, followed by more specific questions regarding impact on sleep and activity.

Interviews were performed by KTB (MD, PhD) and/or CWGA (MD), experienced medical doctors int the department, trained for this study, but not involved in their treatment. All interviews were audio recorded and transcribed verbatim. The transcripts were analyzed inductively by thematic content analysis for categories of intensity descriptors. Data was managed in a framework (case- and theme-based matrix) including specific wording. Coding was done together for 5 transcripts and individually by CWGA and KTB for 5 transcripts, reaching consensus through a meeting comparing assigned codes.

### Clinician interviews

10 clinicians were recruited from Horsens Regional Hospital and from the acute pain service at Aarhus University Hospital, a strategic convenience sample of male/female nurses/doctors working with pain assessment daily. Semi-structured interviews were conducted in Danish, following consent, individually in a private office setting by KTB using a guide (translation in Supplementary data). Transcription and analysis were conducted as for the patient interviews. Coding was done together for 1 transcript and individually by CWGA and KTB for 9 transcripts, reaching consensus through a meeting.

### Patient telephone interviews

100 orthopedic day surgery patients were recruited for telephone interviews conducted 1 week postoperatively. Inclusion was consecutive and dependent on age above 18 years and written informed consent before discharge. 1 patient spoke English, but all other interviews were conducted in Danish. Questions included open-ended inquiries as to pain intensity during the first week, application of the NRS, and comments on the numeric scale. During these interviews, written notes were taken on the interview guide (translation in Supplementary data), as time restraints did not allow recording and transcription. Analysis was again made by coding done independently by CWGA and KTB and reaching consensus.

### Ethics, permissions, funding, and disclosures

This non-interventional study was not applicable for approval by the ethics committee but was approved by our head of hospital. Participants provided informed consent for interviews and recordings. The study received no official grants, but institutional support was received from Horsens Regional Hospital. There were no conflicts of interest. Complete disclosure of interest forms according to ICMJE are available on the article page, doi: 10.2340/17453674.2024.42182

## Results

### Literature

Literature searches were conducted in January 2022, and last updated January 18, 2023. Included for text analysis were: 1 review (9), its references containing pain measures (n = 38), and other pain measures obtained partly through these, partly through 2 related reviews [21,22], and from the Google image search (Figure). The extracted and categorized wording is displayed in Table 2 (see Appendix). Other relevant wording, which did not directly describe intensity, pertained to pain relief (e.g., better/worse), timing (e.g., constant/transient), present, anticipated, or recalled (worst/least/average/usual), or circumstances (at rest/when coughing/during movement).

**Table 2:** Wording for description of pain intensity derived from the literature.

Intensity
No pain (sensation/at all), absence of pain, pain free, none, no hurt, least possible pain, barely perceptible, just/hardly noticeable, extremely weak, very weak, weak, very mild, little, mild, slight, hurts a little bit, faint, a little bit, minor pain, hurts a little more, low, not at all severe moderate, barely strong, some, painful, hurts even more, middle levels, somewhat, quite a bit, considerable, sore, hurting, aching, tender, slightly moderate, very moderate, slightly intense, fairly severe, strong, the pain is bad, high, severe, intense, hurts a whole lot, marked pain, very strong, very severe, very much, very intense, tremendous, fulminant, colossal, giant, maximum pain, maximal amount of pain, most severe (pain possible), pain which could not be more severe, hurts worst, hurts the most, pain as bad as it could be, worst, worst pain (possible/ever/imaginable/experienced), pain as bad as you can imagine, most intense pain (sensation) imaginable, worst imaginable pain, worst pain possible, extremely strong, extremely intense, extremely severe, worst pain ever (experienced/felt), extreme pain, as bad as it could (possibly) be, most, the most severe pain you can possibly imagine, pain cannot be worse, unspeakable, unimaginable, excruciating
**Evaluative** Negligible pain, not significant pain, comfortably manageable, acceptable, bearable, just bearable, unbearable, tolerable, tolerable with discomfort, intolerable, satisfactory, unsatisfactory, troublesome, increasing burden, excessive, exaggerated, uncontrolled, can’t bear the pain
**Impact on cognition** Ability to think and make decision, distracting, (sometimes) distracts me, annoying enough to be distracting, overwhelming, can easily be ignored/hard to ignore/cannot be ignored, can be ignored if you are really involved in your work, but still distracting, can’t be ignored for more than 30 min., can’t be ignored for any length of time, pain bothers me but I can ignore it most of the time, patient able to adapt psychologically, patient unable to adapt pain, interferes with concentration, makes it difficult to concentrate, pain is all I can think about, hard to think of anything else, think about pain all the time, think of pain most of the time, constantly aware of pain, focus of attention, low level: only aware when I pay attention to it, blinding, nothing else matters
**Impact on activity** Interference with some/most activities, inability to do any activity, interference with activity/walking/work, interference with activities in/out of bed, does not interfere with everyday activities, does not interfere with most daily living activities, no effect on ordinary activity but pain after unusual activity, can do usual activities, interrupts some activities, avoid usual activities, prevents doing daily activities, interferes significantly with daily living activities, unable to perform daily living activities, unable to engage in normal activities, limits activity, I’m active but have had to make some modifications or give up some activities because of pain, does not interfere with activities, keeps me from most activities, I give up many activities due to pain, physical activity severely limited, can’t do some daily activities, can continue most activities, you can still go to work and participate in social activities, hard to do anything, unable to do anything, can’t do anything because of pain, pain keeps me from doing most of what I need to do, can do most things, but pain gets in the way of some, can do everything I need to do, slight/moderate/severe pain on movement, interference with movement, moving appropriately or not, interference with mobility/movement, unable to move, you can still function with effort, you can read and converse with effort, unable to speak, can barely talk or move, talking and listening are difficult, interferes with all tasks except taking care of basic needs such as toileting and eating, requires lifestyle changes but patient remains independent, interference with coughing/breathing deeply, paralyzing, disabling: Patient is disabled and unable to function independently, rest or bedrest required, totally disabled - wheelchair or bedridden, unconscious: pain makes you pass out
**Impact on sleep** Interferes with sleep, interference with falling/staying asleep, were you woken up by pain last night, awake with pain most of the night, awake with occasional pain, normal sleep
**Associated symptoms** Tiring, exhausting, nauseating, sickening, suffocating, nausea and dizziness set in as factors of pain, crying out or moaning uncontrollably near delirium, hallucinatory
**Treatment** Desire for opioids, requesting pain relief, threshold for treatment, preference for treatment, requires intervention, what treatments are you receiving, I manage without painkillers, painkillers give complete/moderate/very little/no relief, to what degree do you rely on pain medication or pain- relieving substances for you to be comfortable? (VAS none-some-all the time), no medication needed, mild painkillers are effective (aspirin, ibuprofen, tylenol), mild painkillers relieve pain for 3-4 hours, mild pain killers reduce pain for 3-4 hours, stronger painkillers reduce pain for 3-4 hours (codeine, Vicodin), stronger painkillers are only partially effective, strongest painkillers relieve pain (oxycodone, morphine), stronger painkillers are minimally effective, strongest painkillers reduce pain for 3-4 hours, strongest painkillers are only partially effective, inadequate pain control, effective, just about right, would like to reduce medication, able to adapt with medication or devices such as cushions, uses aspirin or similar medication, requires stronger medication than aspirin or similar medications, requires pain medicine frequently, need emergency help
**Affective** Aggressive, agonized(face), agonizing, anguishing, annihilating, annoying, awful, brutal, concern, cruel, damn, demonic, depressing, desperate, destructive, diabolic, disastrous, discomforting, distressing (slightly, moderately, very), disturbing, dreadful, emotional impact – anxious/depressed /frightened/helpless (NRS 0–10 not at all-extremely), excruciating, fearful, frightening, frightful, grueling, happy face, harmful, hateful, horrible, importunate, inhuman, irritating, killing, miserable, monstrous, nagging, oppressive, prejudicial, punisher, punishing, ravage, terrible, terrifying, tormenting, torturing, unbridled, uncomfortable, unfortunate, unpleasant, upsetting, utterly horrible, very distressing, vicious, violent, wretched
**Discriminative** Aching, beating, boring, burning, cold, compressing, cool, cramping, crushing, cutting, deep, drawing, drilling, dull, flashing, flickering, freezing, gnawing, grinding, heavy, hot, itching, itchy, jumping, lacerating, lancinating, numb, numbing, penetrating, piercing, pinching, pounding, pressing, pricking, pulling, pulsing, quivering, radiating, rasping, scalding, searing, sharp, shooting, smarting, splitting, spreading, squeezing, stabbing, stinging, taut, tearing, throbbing, tight, tingling, tugging, twinges - minor/strong (sudden sharp localized pain), wrenching
**General** Hindering recovery, no functional interference, optimal levels for maximizing functional capacity, interference with mood/relations/enjoyment

**Figure:**
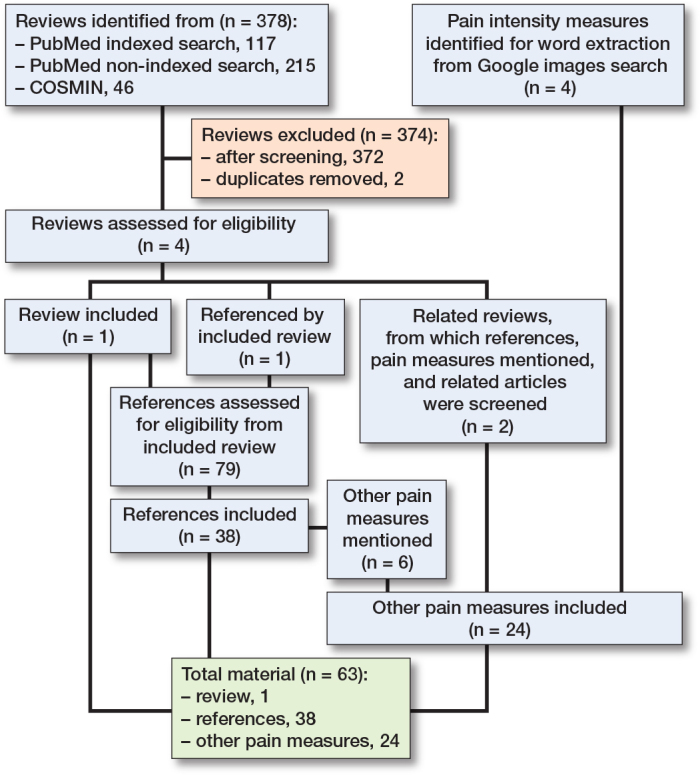
Flowchart for pain intensity manuscripts and measures included in the analysis.

In the Google search for images, identified elements were: blank line (VAS), marked ruler with numbers (NRS) and/or written descriptors, boxes, faces, table, colors (many variations but often green–yellow–orange–red), shades (white-to-black or more intense red), speedometer, wedge, staircase, thermometer, and lines/columns/circles of increasing size. Each step or number on the scale was not necessarily marked by a face or word, making it possible to choose an answer in between. Scales were horizontal or vertical and low-to-high or high-to-low.

### Patient on-site interviews

Interviews were conducted from November 2021 to March 2022. Patients were all literate and ethnically Danish, 3 males and 7 females, all interviewed postoperatively on the day of their surgery. Age was 33–74 years (mean 52.5 years), and they were a broad representation of orthopedic day surgery patients (hand, foot, hip, knee, and shoulder). At the time of interview, their NRS pain scores were: 0, 1, 3, 3, 4, 4, 5, 5, 5-or-6, and 7.

When asked what their pain intensity score meant to them, most patients described it in terms categorized as intensity (translated: It is not sore as such, I can feel it/moderate/it hurts), evaluative (tolerable/I can live with it), and discriminative (radiating/shooting/constricting). When asked directly, patients could describe how their pain would affect activity and their ability to sleep, and whether they needed additional analgesics. Only higher pain levels involved terms categorized as affective (NRS7: irritating, NRS5–6: It is not [intense enough to be] annoying), physical signs (It’s not like I’m lying here sweating), or with cognitive impact (inability to abstract from the pain).

A large variation in the 2 descriptions of the pain score “5” was found: 1 patient could easily accept, move, and possibly sleep with the pain, and another could do none of these. Also, 1 patient labelling her pain as NRS 3 described being pain free at rest, but with NRS 7 when moving.

Several found it easier to describe pain intensity in relation to previous experiences like stubbing a toe on a hard surface, giving birth, known chronic pain elsewhere (e.g., back pain) or the pain they had before surgery.

Overall, the categories induced from the patient interviews were: intensity, evaluative, discriminative, cognitive impact, sleep impact, activity impact, treatment, affective, exemplification, and physical signs. Furthermore, rest/movement, localization, timing (constant/varying), and recent pain medication were important variables to consider.

### Clinician interviews

4 anesthesiologists (3 male) and 6 female nurses were interviewed from November 2021 to January 2022. Notably, in the clinician interviews the category of physical signs was elaborated (e.g., cold sweating/rising blood pressure and pulse/grimacing).

When asked to describe the NRS in words, all but 1 chose spontaneously to group the numbers by intensity (e.g., mild/moderate/severe) as describing each individual number was difficult.

No discriminative wording was used. Exemplification was used frequently. Differences between interviewed clinicians were smaller than between the interviewed patients, possibly reflecting similar training.

Several clinicians mentioned discrepancy between patients’ use of the NRS and clinical observations. For example, patients saying “10” when they can talk and move without difficulty. This discrepancy led the clinicians to suspect that the patients did not interpret the scale in the same way as they did.

### Patient telephone interviews

100 patients (47 male) were telephoned 1 week after surgery from January 2023 to March 2023. Age was 19–80 years (mean 50.8 years), and they were a broad representation of orthopedic day surgery patients (hand, foot, hip, knee, and shoulder). Their expressions of pain intensity fell within the categories previously identified, thus no new categories were produced. *Intensity:* Swearing (only high intensity) and many negatives (not so bad) were observed. *Activity:* Both a way of describing intensity (I couldn’t walk due to pain), and a cause of pain (I hurt because I walked too much). *Sleep impact:* 26 patients mentioned impact or lack of impact on sleep. *Discriminative:* few but frequent words (burning/throbbing/stinging/pressing/pin pricks/jolts). *Treatment:* they described pain intensity by which treatment they used, and how well it worked: medication as well as elevation, ice, compression, and exercises to reduce swelling. 1 patient suggested the toe be amputated, to express severe intensity. When describing their medication, some patients also described their use being reluctant (by principle or side effects) or preventative (by instructions rather than pain). *Evaluative:* some local vocabulary and culturally stereotypical modesty/underplayed expressions (not worth mentioning). *Affective:* wording such as: not too bothered by it, hurt like hell. *Cognitive:* Only 4 patients used wording in this category, such as: I was able to abstract from it and watch a whole movie. *Examples:* only bruising was mentioned twice, otherwise examples were unique. *Physical signs:* Some would describe the extent of swelling and bruising to convey their degree of pain, some mentioned crying or almost crying, one said: It’s not like I’m lying down writhing. *Associated symptoms:* Many other symptoms were mentioned, but not related to pain intensity except: I was sick from the pain. *General recovery:* I am well/I feel good. Other descriptions than intensity or impact were about localization or timing (in the mornings/comes-and-goes/continuing).

A full table in Danish with wording from the 10 transcribed patient interviews, clinician interviews, and telephone patient interviews is available from the authors on request. To assess inductive thematic saturation, a saturation table was made for the coded categories (Table 3).

**Table 3:** Saturation table

Categories	Where category appeared			
	Literature	Patient interviews	Clinician interviews	Patient telephone interviews
Intensity	X	X	X	X
Affective	X	X	X	X
Evaluative	X	X	X	X
Cognitive impact	X	X	X	X
Sleep impact	X	X	X	X
Activity impact	X	X	X	X
Treatment	X	X	X	X
Discriminative	X	X		X
Associated symptoms	X			X
General recovery	X			X
Exemplification		X	X	X
Physical observations		X	X	X

## Discussion

The aim of this study was to perform the first step in creating a new measure of acute postoperative pain, with a defined construct, target population and context of use, namely concept elicitation.

We showed that there are 12 categories for describing acute pain intensity, and we have established vocabularies in English (from the literature) and Danish (from interviews of patients and clinicians). Also, graphic elements have been identified for consideration in the design of a new measure. We have identified issues that must be considered when measuring: timing (current/usual/worst), recent treatment, reluctance/inclination towards medication, rest/activity, localization and concomitant pain, variation of intensity (constant/twinges), and expectations.

It is important to consider the theoretical basis of the construct being measured. We have clearly defined the construct of interest as the sensory intensity of acute postoperative pain. By repeating measurements, the variation and duration of pain are also obtained. This construct is theoretically based on an at least partial ability of patients to consider the sensory, nociceptive intensity of acute pain separately from emotional impact or coping skills when asked directly (in analogy: the music volume as opposed to any dislike of the music). The sensory-discriminative and affective-motivational components were described in 1968 by Melzack and Casey [23], tested by Gracely et al. [24], and are still relevant in modern neuroscience [25]. The terminology of the International Association of the Study of Pain (IASP) describes pain as being a sensory and emotional experience [26]. Also, the ICD-11 classification of chronic pain is based on the theory that pain severity can be graded based on pain intensity, pain-related distress, and functional impairment [27]. This focus on the sensory experience (“music volume”) does not mean that words from other domains are not involved. To the contrary, many expressions of functional impairment were applied by the patients and clinicians as ways to describe pain intensity. We do not consider the induced categories to be different domains, in the way that sleep quality and social participation are different domains in assessment of depression. The question is: “How intense is your pain?”, and the response is: “So intense I can’t sleep or walk”. Keeping such categories in the library will likely improve the intensity scale rather than confuse the patients, but this remains to be explored.

Another pain measure under development [28] has a much wider population, context of use, and construct of interest. The QUALITE-Pain measure aims to apply to both acute and chronic pain intensity, and to pain from all causes, based on a first phase of 44 interviews.

### Strengths

We have sampled the target population, so both timing (present pain or recalled 1 week) and surgery (orthopedic) are relevant for the intended use of the scale. Patients were not interviewed regarding hypothetical or much earlier recalled pain, which would make the collected data more reliant on imagination, previous experience, or memory. For the next phases, where the specific wording must be selected and the scale designed and validated, it is still essential to involve the specific target population defined by language and surgery as well as linguistic expertise. As Danish literature is scarce in comparison, English literature was chosen, and naturally interviews were in Danish. The two categories “exemplification” and “physical observations” obtained by interviews are not likely unique to Danish patients and clinicians. The quality of measurement is greatly dependent on the scale being understood as uniformly as possible by all trial patients. The categories are likely to be widely applicable, but upcoming studies will determine how broad a population can reliably deal with the same wording.

### Limitations

A qualitative study depends on the ability of the researchers to interview comprehensively and catch relevant codes from the collected information. We optimized this by using interview guides, training for this study, and coding the data mostly separately, so our individual impressions of both the data and coding were applied and discussed. Also, we are as surgeons trained and experienced in communication and pain assessment, particularly with this patient group. The use of different sources (literature, patients, and clinicians) improves coverage. Repetitive findings and no new categories during the 100 telephone interviews are strong indications that we have sufficient data to catch all categories (thematic saturation). It is questionable whether all thinkable words or examples describing pain intensity can ever be listed, but based on our extensive material the identification of relevant wording in the upcoming selection and validation studies is quite certain. Second, we did not interview patients below 18 years due to rules of consent, and coverage for adolescents remains to be studied. Also, although we recruited clinicians of different sex, professions, and workplace, the narrow variation in answers could, in addition to similar training, indicate the need for wider sampling. Another limitation is that the relative importance of the findings is difficult to determine: it is not yet established which category, phrase, or graphic element is most useful.

### Conclusion

Acute postoperative pain intensity is described in a large variety of terms collected and categorized in this study. Many graphic elements may also aid in improving standardized self-report, and we have identified variables important to consider when measuring pain.

### Perspective

These results will be the basis for the next phase in PROM development, which is content validation: identification of the specific wording and graphics most relevant, understandable, and with sufficient coverage.

### Supplementary data

The translated semi-structured interview guide is available on the article’s homepage, doi: 10.2340/17453674.2024.42182

## Supplementary Material



## Notes

KTB was responsible for the conception and design of the study and made the initial draft of the article. KTB and CWGA contributed to the collection, analysis, and interpretation of data, discussed the results, and revised the article critically for important intellectual content, and gave final approval of the version to be published.

The authors gratefully acknowledge Connie Timmermann, associate professor at the University of Southern Denmark and Odense University Hospital, for her assistance with the qualitative methodology.

Handling co-editors: Li Felländer-Tsai

*Acta* thanks Johan Creutzfeldt and Anne Lübbeke for help with peer review of this manuscript.
