# Supplementary material for: Measurement of acute postoperative pain intensity in orthopedic trials: a qualitative concept elicitation study
Source: Acta Orthop. 2024 Nov 7;95:625–32. doi: 10.2340/17453674.2024.42182 (PMC11541802; doi:10.2340/17453674.2024.42182)
Supplement: Supplementary file 1 [file ActaO-95-42182-s1.pdf]

Interview guide for patients on-site

Briefing:

Presentation: My name is Karen Bjørnholdt and I am a physician. I am currently working on a research project where I interview patients dealing with postoperative pain. The purpose of the interview is to find out how you would describe the intensity of your pain. The interview takes approx. 15 minutes The conversation will be recorded to support my memory and ensure that everything is included. The recording is anonymous and will be deleted after 1 year. Participation is completely voluntary. Is this something you would like to participate in?

Interview questions:

On a scale from 0 to 10, how intense is your pain where you have had surgery?

What does [mentioned pain score] mean to you?

How would you describe the intensity of your pain in words?

Is your pain so intense that it affects your sleep?

Is your pain so intense that it affects your activities?

Do you need pain medication at your current pain level?

Which of the following do you think best communicates your pain level?

1. words like mild/severe
2. how it is experienced (bearable/unbearable)
3. whether you are able to sleep/do everyday activities or
4. whether you need more paracetamol/morphine

Do you have anything else you would like to add?

Debriefing:

We are almost done. Thank you very much for your help. If you would like, we can let you know the results of the project (e-mail, feedback on whether I have understood you correctly?)

Interview guide for clinicians

Briefing:

Presentation: My name is Karen Bjørnholdt and I am a physician. I am currently working on a research project that focuses on postoperative pain. The purpose of the interview is to find out how you perceive the pain scale from 0-10. It takes approx. 15 minutes The conversation will be recorded to support my memory and ensure that everything is included. There is also a check-off form that I will ask you to fill out. It is anonymous and will be deleted after 1 year. Participation is completely voluntary. Is this something you would like to participate in?

Research questions:

What do the numbers on the pain scale mean to you?<br>How would you describe the intensity of the pain in correlation to the numbers?	
0	
1	
2	
3	
4	
5	
6	
7	
8	
9	
10	
Which numbers correlate to the pain affecting sleep?	Prevents sleep?
Which numbers correlate to pain affecting activity?	Prevents activity?
Which numbers correlate to the need for analgesics?	Strong analgesics?
Which of the following do you think best communicate the level of pain?	1. words such as mild/severe<br>2. how it is experienced (bearable/unbearable)<br>3. whether you are able to sleep/do everyday activities<br>4. whether you need more paracetamol/morphine
Is there anything else you would like to add?	

Debriefing:
We are almost done. Thank you very much for your help. I would like your e-mail so I can contact you and get your thoughts and comments on the final scale once it is completed.

Telephone interview guide (not for recording and transcription, but we take notes during the interview, with words about pain intensity, character, duration, medication – in summary).

<table>
<tr><td>Briefing</td><td colspan="2">Hi, I am Karen Bjørnholdt/Carina Andersen and am calling from Horsens Hospital to follow up on your operation one week ago. Do you have about 5 minutes to talk now?</td></tr>
<tr><td>1.</td><td colspan="2">We have some questions to go through, but first I would like to hear how you are doing after the operation?</td></tr>
<tr><td>2.</td><td colspan="2">How would you describe your pain in the past week? Can you put some more words on that? Can you describe that further?</td></tr>
<tr><td>Categories</td><td colspan="2">Intensity – Evaluative – Affective – Cognitive – Activity – Sleep – Treatment – Discriminative – Associated symptoms – General reconvalescense – Examples – Physical signs</td></tr>
<tr><td>3.</td><td colspan="2">Can you use the scale from 0 to 10 to describe your pain during the week?</td></tr>
<tr><td>4.</td><td colspan="2">Did you have a nerve block or local anesthetic?</td></tr>
<tr><td></td><td>YES: ______________________________
4A. How long did the anesthetic last?

4B. Was the pain properly covered (by pills) when the anesthetic tapered off?</td><td>NO:___</td></tr>
<tr><td>5.</td><td colspan="2">Did you have an appropriate plan for pain treatment – did you know what to do for pain?</td></tr>
<tr><td>6.</td><td colspan="2">Did you need the planned pain medicine – was it fitting, too much or too little?</td></tr>
<tr><td>7.</td><td colspan="2">Do you have anything else, you would like to talk about?</td></tr>
<tr><td>Debriefing</td><td colspan="2">Then we are done. Thank you very much for your help. I will send you a link to a questionnaire with words describing pain, and will ask about your personal opinion about the words, regardless of how much pain you have had. Your answers to the questionnaire will help us to explain the usual "how bad is your pain from 0 to 10", so we are more sure to understand how much pain you have. You must open the mail on a computer or tablet. The link doesn't work properly on a phone. It is really great that you want to participate! Goodbye.</td></tr>
</table>
